# Avulsion fracture of the anterior superior iliac crest following autograft for anterior lumbar fusion: case report and literature review

**DOI:** 10.3389/fsurg.2024.1327028

**Published:** 2024-01-24

**Authors:** Chao-Yuan Ge, Liang Dong, Zheng-Wei Xu, Wen-Long Yang, Li-Xiong Qian, Xiao-Wei Yang, Ding-Jun Hao

**Affiliations:** Department of Spine Surgery, Honghui Hospital, Xi'an Jiaotong University, Xi'an, Shaanxi, China

**Keywords:** avulsion fracture, iliac crest, donor-site complications, spinal fusion, case report

## Abstract

Avulsion fracture of the anterior superior iliac crest (ASIC) following autogenous bone grafting for anterior lumbar fusion (ALF) is an extremely rare complication. We describe a very rare case of avulsion fracture of the ASIC following autograft for ALF in a revision surgery for treating lumbar tuberculosis. A 68-year-old woman with lumbar tuberculosis underwent posterior debridement and posterior iliac crest bone graft fusion; however, her lumbar tuberculosis recurred 9 months after surgery. She then underwent a lumbar revision surgery, including removal of the posterior instrumentation and debridement, followed by anterior L2 corpectomy, debridement, anterior left iliac crest bone graft fusion, and internal fixation. When walking for the first time on postoperative day 3, she experienced a sharp, sudden-onset pain in the anterior iliac crest harvest area. X-ray revealed an avulsion fracture of the ASIC. Considering her failure to respond to conservative treatment for one week and large displacement of the fracture ends, an open reduction and internal fixation surgery was scheduled. Her pain symptoms were significantly relieved after the operation. Although rare, fracture of the ASIC following autograft for ALF should not be ignored. Fracture of the ASIC is usually treated conservatively. Additional surgical treatment is required only when intractable pain fails to respond to conservative treatment or when there is a large displacement of fracture ends that are not expected to heal spontaneously.

## Introduction

Spinal fusion surgery has been widely used for the management of many spinal disorders (1). There are many materials used in spinal fusion, including autograft, allograft, and artificial materials, which have been reported to yield satisfactory fusion rates (2, 3). However, both allograft and synthetic materials have the disadvantages of high cost, immune rejection, and relatively low fusion rate (4). Although autogenous bone grafts at the surgical site could avoid immune rejection, it raises the issue of insufficient supply due to the availability of a substantial quantity and quality of autogenous bone, and due to the simplicity of harvest, the iliac crest has become the most common site for autografting among spine surgeons (5).

However, bone harvest from the autologous iliac crest is not necessarily a problem-free process. Various complications at the iliac crest donor site, including refractory pain, neurovascular injury, infection, hematoma, and bowel herniation, have been reported (6, 7). Among these, avulsion fracture of the anterior superior iliac crest (ASIC) after bone harvest for spinal fusion is a very rare complication and, to our knowledge, only eight cases have been reported to date (8–15), and only one was for anterior lumbar fusion (ALF) (14). Here, we report the second case of avulsion fracture of the ASIC following autograft for ALF in a revision surgery to treat lumbar tuberculosis, which was successfully managed by open reduction and internal fixation.

## Case presentation

### Patient and initial therapy

**Figure 1:**
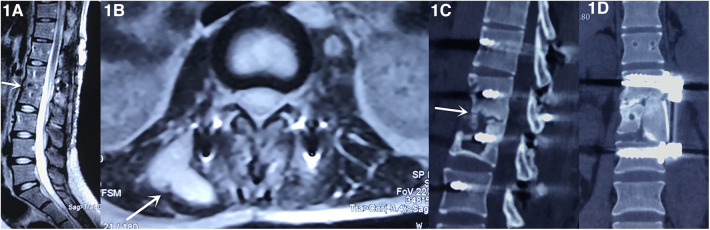
Sagittal T2-weighted magnetic resonance imaging (MRI) of the lumbar spine revealed the presence of pus in the intervertebral space between L1 and L2 (**A**, arrow). Cross-sectional MRI revealed some pus on the right leaking backward under her skin, forming a sinus (**B**, arrow). Sagittal computed tomography (CT) revealed the presence of sequestrum formation at L2, and no bony fusion was achieved in the intervertebral space between L1 and L2 (**C**, arrow). The patient underwent the lumbar revision surgery, including removal of posterior internal fixation and debridement, followed by anterior L2 corpectomy, debridement, autograft bone fusion, and internal fixation (**D**).

A 68-year-old female patient was hospitalized due to a one-month history of back pain, fever, night sweats, and a 10-day history of pus oozing from a lumbar posterior surgical wound. The patient weighed 45 kg and had osteoporosis. She was diagnosed with lumbar tuberculosis and underwent posterior debridement and posterior iliac crest bone graft fusion 9 months prior. She had been taking oral drugs, including isoniazid, rifampicin, pyrazinamide, and ethambutol for antituberculosis treatment, as instructed since her last discharge. On physical examination, a sinus had formed in her lumbar wound through which pus oozed. Severe tenderness at her lumbar wound was noted. Laboratory investigations revealed an elevated erythrocyte sedimentation rate [106 mm/h, (normal range 0‒20 mm/h)] and C-reactive protein [82 mg/dl, (normal range 0‒8 mg/L)] level. Sagittal T2-weighted magnetic resonance imaging (MRI) of the lumbar spine revealed the presence of pus in the intervertebral space between L1 and L2 (Figure 1A). Cross-sectional MRI revealed some pus on the right leaking backward under her skin, forming a sinus (Figure 1B). Sagittal computed tomography (CT) revealed the presence of sequestrum formation at L2, and no bony fusion was achieved in the intervertebral space between L1 and L2 (Figure 1C). The patient was diagnosed with recurrent lumbar tuberculosis and, accordingly, underwent lumbar revision surgery, including removal of posterior internal fixation and debridement, followed by anterior L2 corpectomy, debridement, autograft bone fusion, and internal fixation (Figure 1D). The autograft bone materials were two tricortical blocks harvested with an osteotome using an anterolateral approach to the anterosuperior margin of her left ASIC, as she was fixed in the right lateral decubitus position during surgery. Considering that the bone defect area after lesion debridement was large, the 2 tricortical cortical blocks were also large. One block was about 4.5 cm in length, 2 cm in width and 2 cm in height, the other block was about 4 cm in length, 2 cm in width and 2 cm in height. The revision surgery was successful.

### Diagnostic and surgery

**Figure 2:**
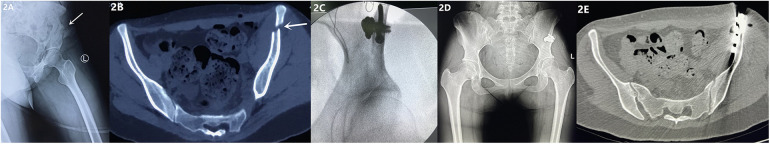
X-ray revealed an avulsion fracture of residual bone in the anterior superior iliac crest (ASIC) (**A**, arrow), which was further confirmed on CT (**B**, arrow). Intraoperative fluoroscopy showed a good reduction of the fracture of ASIC had been achieved by an open reduction and internal fixation surgery (**C**). The postoperative AP x-ray of the pelvis showed a good position of the bone block and the internal fixation (**D**). The CT showed the fracture ends healed well one year postoperatively (**E**).

When walking for the first time on postoperative day 3, she experienced a sharp, sudden-onset pain in the left ASIC harvest area. On physical examination, a mobile subcutaneous mass, approximately 3 cm in diameter, located approximately 3 cm caudal to the bone donor area, was palpated. X-ray revealed an avulsion fracture of residual bone in the ASIC (Figure 2A), which was further confirmed on CT (Figure 2B). The fracture belonged to type A1 according to the AO pelvic fracture classification (16). She was managed with analgesics and activity restriction; however, her pain symptoms did not abate one week later. Considering the failure of conservative treatment for one week and the large displacement of the fracture site, an open reduction and internal fixation operation was scheduled. An incision was made along the original surgical incision, we saw a piece of dissociative bone with a diameter of about 3 cm torn off from the autogenous bone extraction area. After cleaning the fracture end and blood clot, the bone block was drilled by Kirschner wire on both sides, then was fixed with intramedullary nail. Then a bone plate was attached to the iliac bone block after pre-bending, fixed with 4 screws. Intraoperative fluoroscopy showed a satisfactory fracture reduction was achieved (Figure 2C).

## Results

Her pain symptoms were significantly relieved after surgery. The postoperative AP x-ray of the pelvis showed a good position of the bone block and the internal fixation (Figure 2D). The CT showed the fracture ends healed well one year postoperatively (Figure 2E).

## Discussion

As a fusion material, autologous bone graft has become the gold standard for all spinal fusion surgeries due to its excellent properties of osteoconduction, osteoinduction, and osteogenesis (9). The anterior or posterior iliac crest is the most common donor site used by spinal surgeons. It not only provides high-quality cancellous and cortical bone grafts with substantial quantity, but is also convenient for harvest regardless of the patient's position during spinal surgery (5).

Some literatures including *in vivo* and cadaveric research have reported on the amount of bone graft that could be harvested from the iliac crest. In Conway's study, the volume of bone graft could be harvested from anterior iliac crest was 5–72 cm^3^ and 25–88 cm^3^ from the posterior iliac crest, and he believed that the bone quality largely depends on the patient's bone quality (17). A Systematic review conducted by Villarreal-Villarreal in 2023 showed that previous clinical studies could harvest 5.5–17 cm^3^ of bone graft from anterior iliac crest, and cadaveric study could harvest 4.98–69.56 cm^3^ of bone graft from anterior iliac crest (18). A 2023 latest study about quantitative volumetric measurements of bone grafting sites on computed tomography scans also carried out by Villarreal-Villarreal et al. showed that the measured volume was of 19.35 ± 4.16 cm^3^ and 32.48 ± 4.49 cm^3^ for anterior and posterior iliac crest (19). Kilinc et al. used 3-dimensional CT and software in a living adult population, they concluded total of corticocancellous bone that could be harvested was 26 cm^3^ at the widest limit and 19 cm^3^ at the narrowest limit from the anterior, and 34 cm^3^ from the posterior, iliac crest (20).

However, complications in the iliac crest donor area are not uncommon. These complications include wound infection, non-healing, hematoma, refractory pain, intestinal hernia, neurovascular injury, spinopelvic dissociation, iliac wing fractures, and pseudoaneurysm of the deep circumflex iliac artery, which are reported to occur at a rate of 10%–60% (6, 21–23). To date, more than 50 cases of iliac crest fractures after bone harvest have been reported, of which 28 were in the anterior part of the iliac crest (24). Among them, fracture of the ASIC harvested for spinal fusion surgeries is extremely rare; to our knowledge, there have been only 8 reports to date (8–15), as shown in Table 1. Of the 8 reports, only one reported avulsion fracture of ASIC occurred following ALF, the rest of them were all for cervical surgery. Unlike cervical spine fusion, which requires less autologous bone, anterior lumbar reconstruction not only requires more autologous bone, but also requires bicortical or tripcortical bone to provide support. In 2002, Farage et al. first reported the use of titanium mesh for intervertebral bone graft support to reconstruct the anterior spinal column for the treatment of spinal tuberculosis (25). The titanium mesh has a simple structure and has a good biocompatibility and strong support strength. The granular bone fragments filled in the titanium mesh could be fused and grown through the bone graft contact interface. However, tuberculosis lesions usually vary in size, and the bone defects after lesion removal are irregular. Larger titanium meshes may be at risk of damaging the dura mater and nerve roots during placement, while smaller titanium meshes are at risk of loosening, shifting and settling, especially for patients with osteoporosis. On the other hand, autologous iliac bone has the advantages of easy construction and high fusion rate, so we used autologous iliac bone transplantation for the patient.

**Table 1:** The literature review of fracture of anterior iliac crest after autogenous bone grafting for spinal fusion surgery.

References	Age/Sex	Osteoporosis	Diagnosis	Spinal fusion levels	Therapy
Reynolds et al. (10)	52/F	Yes	Cervical radiculopathy	ACDF C5-C6, C6-C7	2 Weeks of bedrest
Reale et al. (11)	51/F	Yes	Cervical radiculopathy	ACDF C5-C6, C6-C7	15 Days of bedrest
Porchet et al. (12)	56/M	Yes	Cervical radiculopathy	ACDF C5-C6, C6-C7	1 Day of bedrest
Al-Sayyad et al. (8)	67/F	Yes	Cervical radiculopathy	ACDF C5-C6	Assisted mobilization with crutches
	61/F	Yes	Cervical myelopathy	ACDF C4-C5	Bedrest followed by assisted mobilization
	63/F	Yes	Cervical radiculopathy	ACDF C5-C6, C6-C7	Symptomatic treatment and gait training using a walker
Ovalioglu et al. (9)	63/F	Yes	Cervical radiculopathy	ACCF C5 and C6	Analgesics and activity restriction
	NG	NG	Lumbar burst fractures	ALF L2-L4	Conservative treatment
Chang-Nam Kang et al. (14)	70/F	Yes	Cervical myelopathy	ACDF C3-C4	Open reduction and internal fixation surgery
Kawaoka et al. (13)	77/F	Yes	Cervical myelopathy	ACDF C3-C4, C4-C5	Open reduction and internal fixation surgery
	80/F	Yes	Cervical myelopathy	ACDF and laminectomy C3-C6	Minimized movement
Burkhardt BW et al. (15)	NG	NG	Cervical disc herniation	ACDF NG	Osteosynthesis surgery

ACDF, anterior cervical discectomy and fusion; ACCF, anterior cervical corpectomy and fusion; ALF, anterior lumbar fusion; NG, not given.

The main mechanism involved in the fracture of the ASIC is related to the attachment of muscles in this area. Unlike oral and maxillofacial surgery and anterior cervical fusion, ALF usually requires significantly larger tricortical bone blocks. When these blocks are harvested from the ASIC, the attachment area of the hip flexors is significantly reduced, and the strength of contraction of the sartorius and tensor fascia latae muscles attached to the ASIC may cause avulsion fracture of residual bone after bone harvesting when the patient starts to walk postoperatively (9). In addition, patient-related factors, such as older age, lower weight, osteoporosis, and anorexia nervosa, have been identified as risk factors for fracture of the ASIC (8, 24). In our case, fracture of the ASIC could be attributed to the patient's older age, low weight, and osteoporosis.

In addition to the above patient-related factors, surgery-related factors, including the area and shape of the bone and the tool used for bone harvesting, also play an important role in treating ASIC fracture. A morphological study of the ilium revealed that the thickest portion of the ilium, located at the iliac tubercle, is 45% thicker than that at a point 30 mm posterior to the ASIC (26). In addition, a biomechanical study investigating the strength of residual iliac crest concluded that the removal of bone from the iliac tubercle maintained 2.4 times more strength compared with that grafted from an area 15 mm posterior to ASIC (27). Thus, the iliac tubercle is the optimal area of bone to harvest from the ASIC (26). In terms of the shape of bone harvested, some researchers have suggested that it is safe to harvest a bicortical bone block rather than a tricortical block, and they believe that the direction of osteotomy is also important. They believe that an oblique, anteriorly angled osteotomy may leave a narrow bone at the bottom, predisposing the ilium to fracture following iliac crest bone grafting (26). Regarding the tools used for bone harvesting, one study concluded that using an osteotome increased the incidence of fracture compared with that using a saw (28). Besides, Ahlmann et al. have suggested that when a larger volume of graft is harvested from the anterior or posterior iliac crest, the donor site morbidity increases (29). In our case, the amount of autogenous bone we harvested from her ASIC was about 34 cm^3^ (4.5 × 2 × 2 + 4 × 2 × 2), exceeding the available amount reported in the literature, so overharvest may play an important role in her fracture. Besides, the partial forward bone graft region, the use of an osteotome and removal of two tricortical blocks, all of these factors contributed to her fracture.

The number and type of complications should be kept in mind whenever bone harvested. In order to avoid fracture of bone harvesting area at ASIC in the future, we deem that its risk factors should be avoided and improved. First, patient selection is very important. Patients with older age, low weight, and osteoporosis were more prone to fracture at ASIC. Autologous bone harvest should be avoided. Allograft bone or titanium mesh or 3D printed prosthesis can be used instead in such patients, or exploration of alternative graft harvesting sites could be taken into consideration. Second, it is important to make a meticulous plan of bone removal before operation, including the area and the estimated size of bone to be harvested. The iliac tubercle is the optimal area of bone to harvest from the ASIC. The estimated size of the bone to be removed is mainly based on the size of the lesion determined by MRI. Third, it is safe to harvest a bicortical bone block rather than a tricortical block. The direction of osteotomy should be vertical, avoiding leaving a narrow bone at the bottom. Besides, we recommend using a saw rather than an osteotome for osteotomy. The process of autogenous iliac bone harvest should be done gently and with great care. In short, fracture of the ASIC after graft harvest can be prevented by careful patient selection, meticulous preoperative planning, exploration of alternative graft harvesting sites and optimal surgical techniques and appropriate tool et al.

Posterior fractures of the iliac crest often lead to significant disability of the pelvic ring, which can be permanent and often require surgical treatment (30). One study reported that 69% of patients with posterior fractures underwent surgical procedures (24). Unlike posterior fractures, despite the pain, fractures of the ASIC usually do not affect the stability of the pelvic ring and can heal spontaneously in most cases without further complications. Therefore, conservative treatments involving a period of rest until the fracture heals is usually recommended for patients who experience anterior fracture (30). It has been reported that 86% of patients with anterior fractures successfully respond to conservative treatment (24). However, the conservative treatment is not risk-free, the most frequent complications are exostosis formation, non-union and persistent pain (31). Occasionally, an anterior fracture requires additional surgical treatment when a patient's intractable pain fails to respond to conservative treatment or in the presence of large displacement of fracture ends that are not expected to heal spontaneously (32), as the case reported in our patient. The traditional surgical method is open reduction and fixation using non-locking reconstruction plates or one-third tubular plates or suture anchors, and its clinical outcome is usually good. Aurich proposed a novel internal fixation using anatomic low-profile locking plate to fix the displaced ASIC, he deemed it has the advantage of non-locking cancellous lag screws and quick and easy application (32).

It is noteworthy that for patients who experience fracture of the ASIC, pain at the donor site incision in the early postoperative period may mask the pain caused by fracture of the ASIC. In addition, some paralyzed patients who undergo cervical or thoracic spinal decompression surgery may lose their pain perception at the fracture site. Therefore, postoperative x-ray of the pelvis and careful physical examination is essential in these patients.

## Conclusion

Avulsion fracture of the ASIC after autograft for ALF is an uncommon complication. Better understanding of graft harvesting techniques and identifying risk factors will be helpful in minimizing this complication. Fracture of the ASIC is usually treated conservatively, and additional surgical treatment is required only when the patient's intractable pain fails to respond to conservative treatment or when there is a large displacement of fracture ends that are not expected to heal spontaneously.

## Data Availability

The original contributions presented in the study are included in the article/Supplementary Material, further inquiries can be directed to the corresponding author.
